# Influence of instrument parameters on the electrochemical activity of 3D printed carbon thermoplastic electrodes

**DOI:** 10.1038/s41598-023-27656-7

**Published:** 2023-01-07

**Authors:** Ricoveer Singh Shergill, Chloe L. Miller, Bhavik Anil Patel

**Affiliations:** 1 School of Applied Sciences, Brighton, BN2 4GJ UK; 2 Centre for Stress and Age-Related Disease, Brighton, BN2 4GJ UK

**Keywords:** Electrochemistry, Sensors

## Abstract

3D printing provides a reliable approach for the manufacture of carbon thermoplastic composite electrochemical sensors. Many studies have explored the impact of printing parameters on the electrochemical activity of carbon thermoplastic electrodes but limited is known about the influence of instrument parameters, which have been shown to alter the structure and mechanical strength of 3D printed thermoplastics. We explored the impact of extruder temperature, nozzle diameter and heated bed temperature on the electrochemical activity of carbon black/poly-lactic acid (CB/PLA) electrodes. Cyclic voltammetry and electrochemical impedance spectroscopy measurements were conducted using standard redox probes. The electrode surface and cross-section of the electrode was visualised using scanning electron microscopy. We found that using extruder temperatures of 230 °C and 240 °C improved the electrochemical activity of CB/PLA electrodes, due to an increase in surface roughness and a reduction in the number of voids in-between print layers. Nozzle diameter, heated bed temperature of different 3D printers did not impact the electrochemical activity of CB/PLA electrodes. However high-end printers provide improved batch reproducibility of electrodes. These findings highlight the key instrument parameters that need to be considered when manufacturing carbon thermoplastic composite electrochemical sensors when using 3D printing.

## Introduction

3D printing as a manufacturing approach has provided the ability to make electrochemical sensors at mass production in varying complex geometries^1–8^. The material used to make 3D printed electrodes contain a fixed percentage of conductive material (e.g., different forms of carbon) which is mixed with a non-conductive thermoplastic such as polylactic acid (PLA). Therefore, all 3D printed electrodes are composite electrodes, where a fraction of the electrode is conductive^9,10^. Historically, carbon composite electrodes have high batch variability^5,11–14^, due to difficulties in making uniform electrodes often by man manufacturing. However, the machine manufacturing of electrodes using 3D printing provides greater precision between batches of electrodes and thus makes this a suitable approach for reproducible manufacturing of carbon composite electrodes^11^.

The printing process can influence the construction of the printed part and thus can alter the electrochemical activity of the carbon thermoplastic composite electrode. The manufacture of 3D printed electrodes can be influenced by printing parameters and instrument parameters. Printing parameters affect the architectural structure of the electrode when printed and instrument parameters are variables that influence the extrusion of the carbon thermoplastic filament. Many studies have focused on exploring the influence of printing parameters, where print orientation, printing speed and layer thickness were shown to alter the electrochemical activity of carbon conductive electrodes^11,15–18^. No studies have investigated the influence of instrument parameters on the electrochemical activity of 3D printed carbon thermoplastic electrodes. However, studies exploring the impact of instrument parameters have been conducted mainly on thermoplastics such as PLA^19–25^, where differences were observed when altering the nozzle diameter, heated bed and extruder temperature. Varying studies have shown that printing parts using larger nozzle diameters enhanced the tensile strength of the printed parts, although it was not linearly correlated^26–28^. It is assumed this may be due to potentially a slight increase in the width of the layer with increasing nozzle diameter. Studies have also shown that the tensile strength of PLA printed parts increase as the heated bed temperature increased. As the heated bed temperature increases, there is an increase in heat dissipation from one layer to another, which leads to post-heating of layers which are already bonded. Due to this post-heating of layers, greater diffusion of one layer to the adjacent layer occurs and hence improves the strength. This enhanced adhesion was significantly increased when printing parts at a bed temperature slightly above the glass transition temperature (Tg) of the printing material^28–30^. Various studies have shown that using higher extruder temperatures improved the tensile mechanical properties of carbon-fiber PLA and PLA. This was attributed to a reduction in the total number of voids present in-between the print layers, which enhances the inter-layer bonding between layers^20,25,28,31–33^.

Studies conducted on PLA have highlighted significant differences in the structure of the electrode with varying instrument parameters, which influenced the mechanical properties of the printed part, however it is not known if these structural changes influence the electrochemical activity of the printed part. Our study explored the impact of instrument parameters on the electrochemical activity of carbon black/poly lactic acid (CB/PLA) electrodes. We made CB/PLA electrodes at varying extruder temperatures, heated bed temperatures and different nozzle diameters. These electrodes were investigated using cyclic voltammetry and electrochemical impedance spectroscopy. The surface area and cross-section of the printed electrodes were characterised using scanning electron microscopy. Finally, we highlighted the implications of our findings in how to optimise the manufacturing of CB/PLA electrodes for sensing applications.

## Results and discussion

### Influence of nozzle diameter on electrochemical activity of CB/PLA electrodes

**Figure 1:**
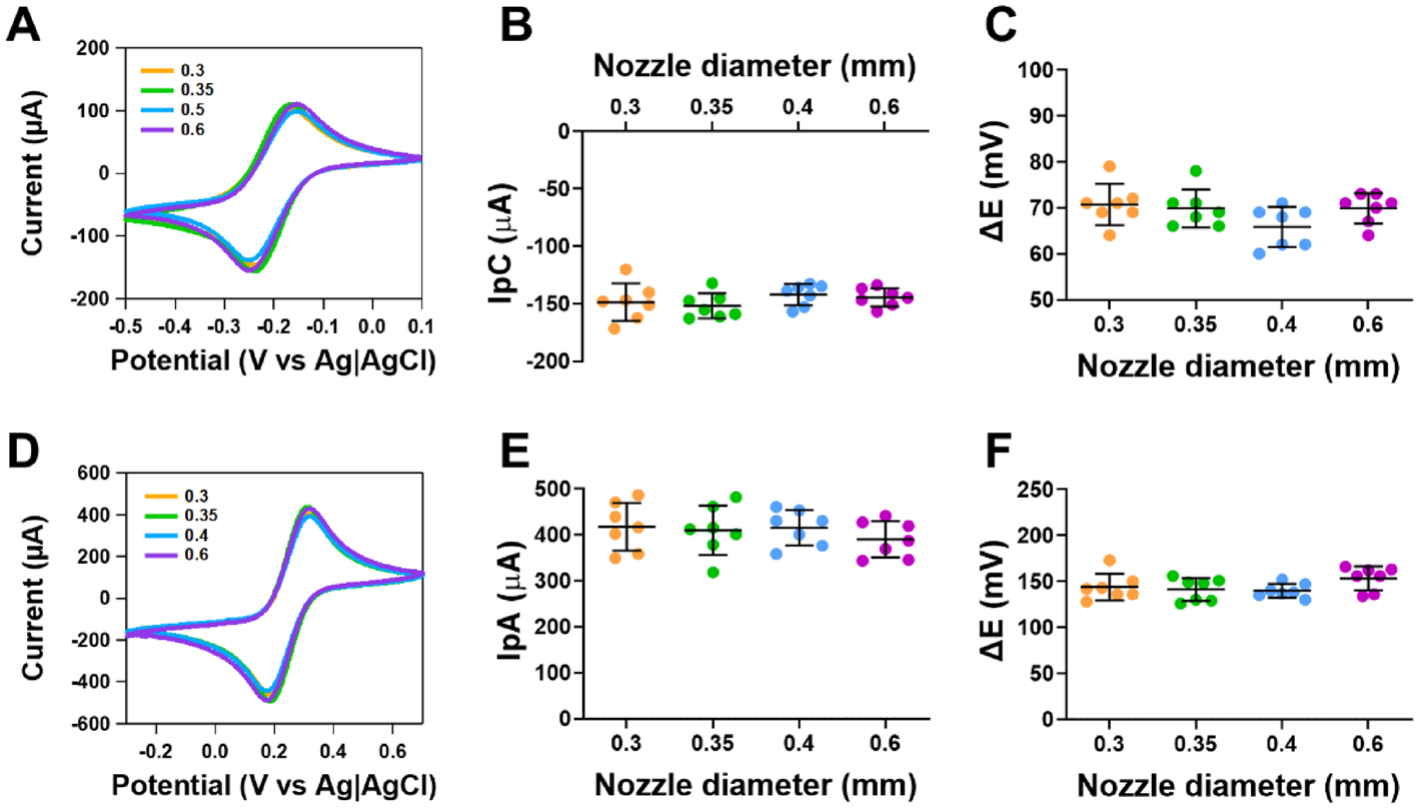
Responses of 3D printed CB/PLA electrodes at different nozzle diameters on outer and inner sphere redox probes. (**A**) Representative cyclic voltammograms of 1 mM ruthenium hexamine in 1 M KCl at 0.05 V s^−1^, (**B**) Cathodic peak current, (**C**) ΔE of ruthenium hexaamine (**D**) representative cyclic voltammograms of 5 mM ferricyanide in 1 M KCl at 0.05 V s^−1^, (**E**) anodic peak current and (**F**) ΔE of ferricyanide. All electrodes were printed at 230 °C extruder temperature and heated bed temperature of 50 °C. Data shown as mean ± SD, where n = 7.

Numerous studies have used different diameters of the nozzles to make CB/PLA electrodes^1,6,34–36^, but it is not known if this influences the electrochemical activity. Using an extruder temperature of 230 °C and a heated bed temperature of 50 °C, we investigated how the nozzle diameter altered the electrochemical activity of CB/PLA electrodes. Figure 1A shows cyclic voltammograms for outer-sphere redox probe ruthenium hexaamine, where no differences in the responses were observed. When comparing nozzle diameters from 0.3 to 0.6 mm, there was no significant difference in the cathodic peak current (n = 7, Fig. 1B) and ΔE (n = 7, Fig. 1C). Figure 1D shows cyclic voltammograms for inner-sphere redox probe ferricyanide, where no differences in the responses were also observed. When comparing between nozzle diameters from 0.3 to 0.6 mm, there was no significant difference in the anodic peak current (n = 7, Fig. 1E) and ΔE (n = 7, Fig. 1F).

Table 1 shows the percentage success rate of making printed parts that had the same geometry of the electrode using either PLA or CB/PLA. For parts made using PLA, there was a 100% success rate across all nozzle diameters. However, for CB/PLA, there was a significant reduction in the success rate with increasing nozzle diameter. Thus, the highest nozzle diameter (0.6 mm) had the greatest success rate for both PLA and CB/PLA. This differential effect is most likely due to the impact of CB particles in the CB/PLA filament, which can easily clog smaller nozzle diameters. Studies have shown that larger nozzle diameters are less prone to clogging and abrasion from the filament during printing over a sustained period, thus allowing for a longer lifespan of use^37,38^.

**Table 1:** Success rate of PLA and CB/PLA parts (using the same geometry of the electrodes) made using varying nozzle diameters, where n = 10.

Nozzle diameter (mm)	PLA part print success rate (%)	CB/PLA part print success rate (%)
0.3	100	20
0.35	100	30
0.5	100	80
0.6	100	90

All printed parts were made at 50 °C heated bed temperature and 230 °C extruder temperature.

These findings highlight that when printing electrodes at the same layer thickness using different nozzle diameters, there is no variation in the electrochemical activity of the electrode, however using larger nozzle diameters increased the success rate for manufacturing electrodes. This may not be the case for different print layer thickness, which can be used to make electrodes, given that there is an upper and lower tolerance for each nozzle diameter.

### Impact of heated bed temperature on the electrochemical activity of CB/PLA electrodes

**Figure 2:**
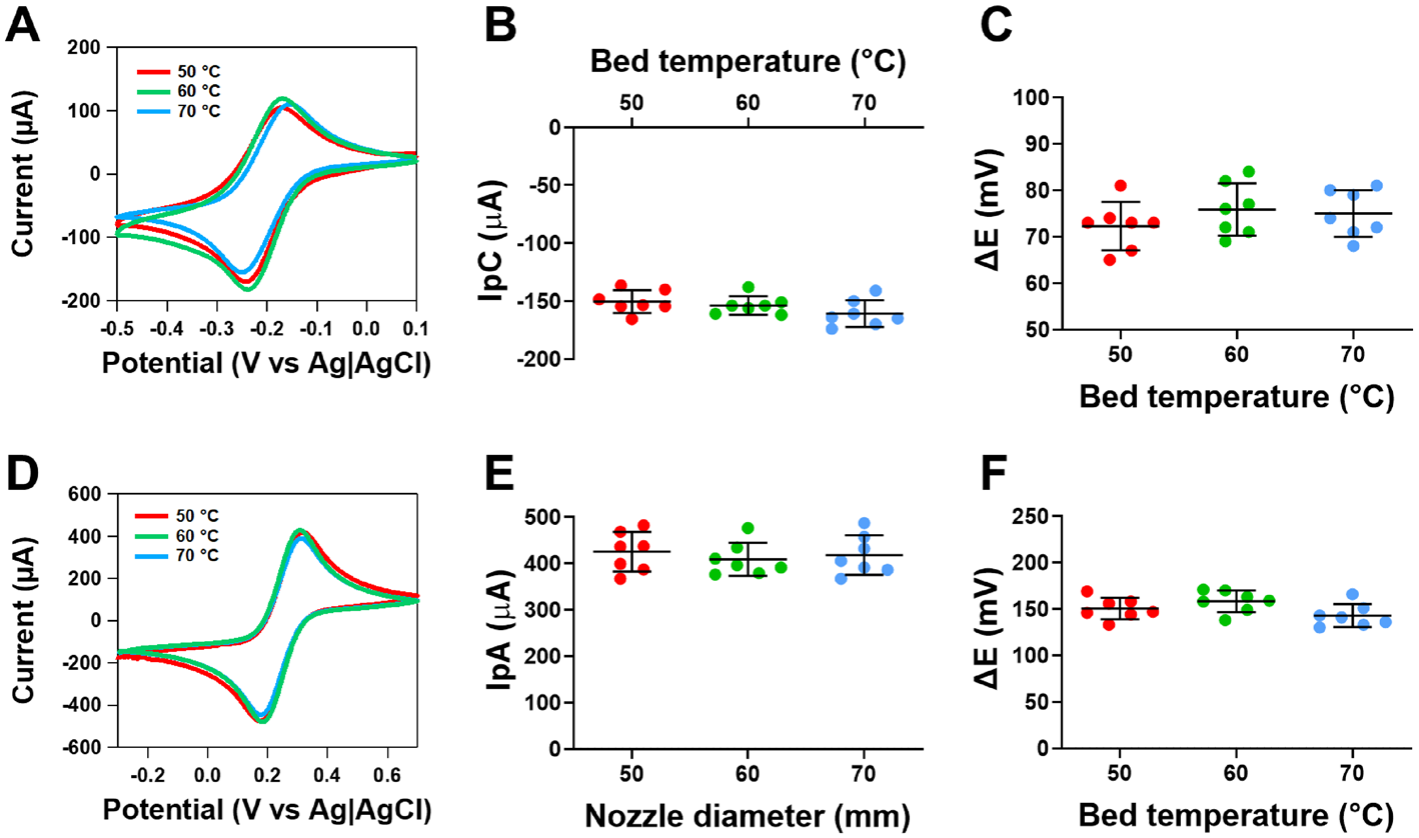
Responses of 3D printed CB/PLA electrodes at varying heated bed temperatures on outer and inner sphere redox probes. (**A**) Representative cyclic voltammograms of 1 mM ruthenium hexaamine in 1 M KCl at 0.05 V s^−1^, (**B**) Cathodic peak current, (**C**) ΔE of ruthenium hexaamine (**D**) representative cyclic voltammograms of 5 mM ferricyanide in 1 M KCl at 0.05 V s^−1^, (**E**) anodic peak current and (**F**) ΔE of ferricyanide. The extruder temperature was 230 °C and the nozzle diameter was 0.6 mm. Data shown as mean ± SD, where n = 7.

Published studies have used heated bed temperatures of 50 and 70 °C^1,11,35,39^. The temperature of 70 °C is above the glass transition temperature but was chosen as studies have shown adhesion between layers increased when utilising a bed temperature slightly above the glass transition temperature of the printing material^29,30^. Figure 2A shows cyclic voltammograms for ruthenium hexaamine, where no differences in the responses were observed. When comparing between the heated bed temperatures, there was no significant difference in the cathodic peak current (n = 7, Fig. 2B) and ΔE (n = 7, Fig. 2C). Figure 2D shows cyclic voltammograms for ferricyanide, where no differences in the responses were observed. When comparing between the heated bed temperatures, there was no significant difference in the anodic peak current (n = 7, Fig. 2E) and ΔE (n = 7, Fig. 2F).

These findings highlight that the headed bed temperature does not influence the electrochemical activity of our 3D printed CB/PLA electrodes, but this is not what is observed for PLA parts, where increased heated bed temperature has shown to improve the adhesion between adjacent layers and thus improves the strength of the electrode^29,30^. In our findings, no difference in the current response was observed, which may be because our electrode was printed in a vertical orientation (as this optimised the conductivity of the electrode). In this orientation, only a small fraction of the electrode may have benefited from the enhanced adhesion from elevated heated bed temperatures. However, electrodes made with very few print layers may be more impacted by headed bed temperature.

### Exploring the changes in electrochemical activity of CB/PLA electrodes made at higher extruder temperatures

**Figure 3:**
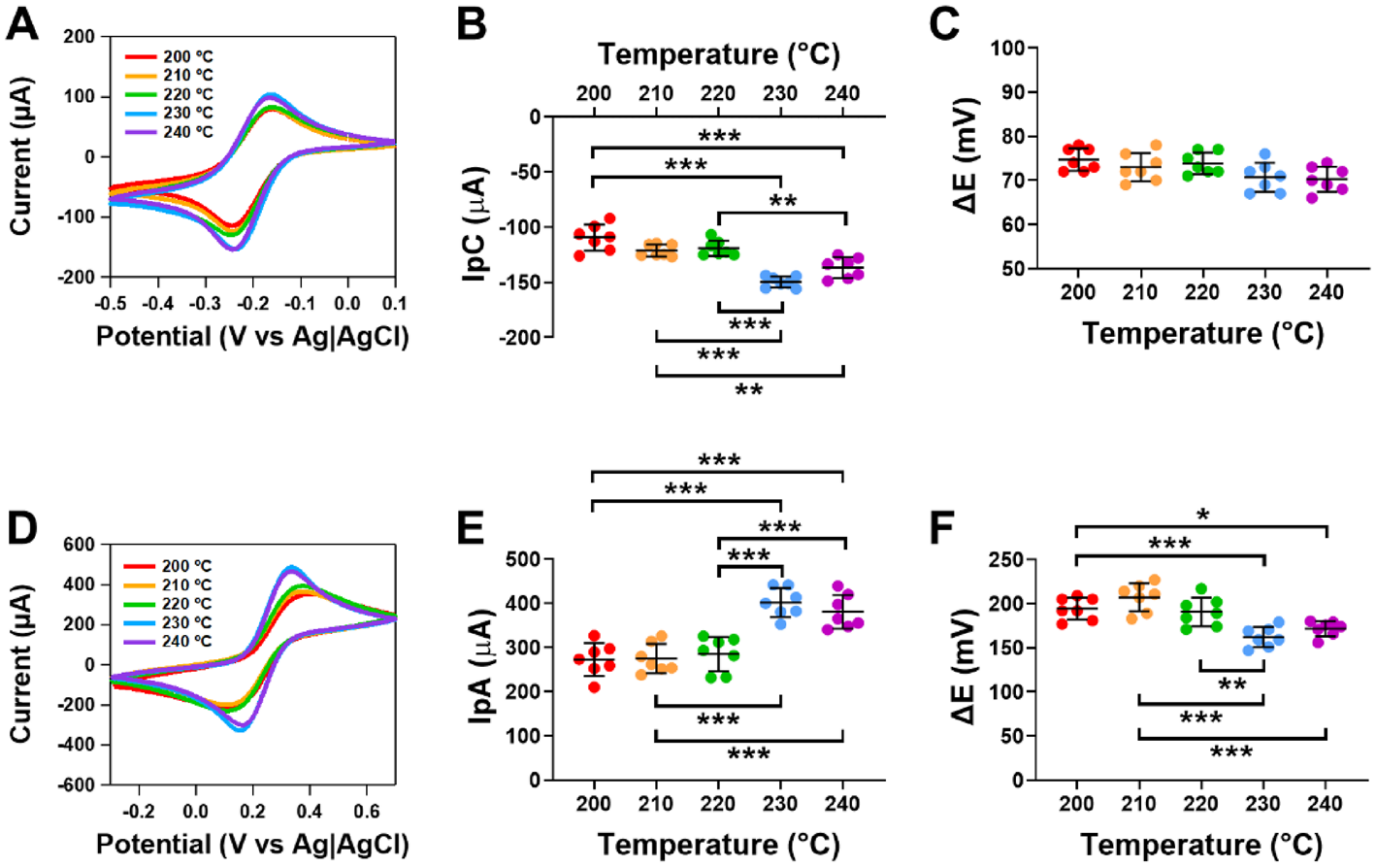
Responses of 3D printed CB/PLA electrodes at different extruder temperatures on outer and inner sphere redox probes. (**A**) Representative cyclic voltammograms of 1 mM ruthenium hexaamine in 1 M KCl at 0.05 V s^−1^, (**B**) Cathodic peak current, (**C**) ΔE of ruthenium hexaamine (**D**) representative cyclic voltammograms of 5 mM ferricyanide in 1 M KCl at 0.05 V s^−1^, (**E**) anodic peak current and (**F**) ΔE of ferricyanide. The nozzle diameter was 0.6 mm and the heated bed temperature was 50 °C. Data shown as mean ± SD, where n = 7, *p < 0.05, **p < 0.01, ***p < 0.001.

Figure 3A shows cyclic voltammograms of the 3D printed electrodes when assessed using ruthenium hexaamine. Figure 3B shows that there was a significant increase in the cathodic peak current for electrodes printed at 230 °C when compared to 200 °C, 210 °C and 220 °C (*p* < 0.001, n = 7). A significant increase in the current was also observed for electrodes printed at 240 °C when compared to 200 °C (*p* < 0.001), 210 °C and 220 °C (*p* < 0.01, n = 7). No difference in the cathodic peak current was observed between electrodes printed at extruder temperatures of 230 °C and 240 °C (n = 7). There was no significant difference in ΔE for electrodes printed at the varying extruder temperatures (n = 7, Fig. 3C).

To further explore the differences between the electrodes made at different extruder temperatures, measurements were conducted using inner sphere redox probe ferrocyanide. Figure 3D shows clear differences in the cyclic voltammograms of ferrocyanide on electrodes made at varying extruder temperatures. Figure 3E shows the differences in the anodic peak current for ferrocyanide, which followed a similar pattern to that observed for ruthenium hexaamine. There was a significant increase in the anodic peak current for electrodes printed at 230 °C when compared to 200 °C, 210 °C and 220 °C (*p* < 0.001, n = 7, Fig. 3E). A significant increase in the anodic peak current was also observed for electrodes printed at 240 °C when compared to 200 °C, 210 °C and 220 °C (*p* < 0.001, n = 7). No difference in the anodic peak current was observed between electrodes printed at extruder temperatures of 230 °C and 240 °C (n = 7, Fig. 3E). The electroactive surface area calculated using the using the Randles–Ševčík equation showed that for the electrode made at extruder temperatures of 230 °C the active surface area was 0.157 ± 0.01 cm^2^, which equates to 20.0 ± 1.6% of the electrode (Supplementary Table 1). Figure 3F shows that ΔE was significantly lower for electrodes printed at 230 °C when compared to 200 °C (*p* < 0.001), 210 °C (*p* < 0.001) and 220 °C (*p* < 0.01, n = 7). A significant decrease in ΔE was also observed for electrodes printed at 240 °C when compared to 200 °C (*p* < 0.05) and 210 °C (*p* < 0.001, n = 7, Fig. 3F). The heterogenous electron transfer kinetics (HET, k°) were calculated based on the method by Nicholson^40^, showed that for the electrode made at extruder temperatures of 230 °C the k° was 5.9 × 10^−5^ ± 9.3 × 10^−6^ cm s^−1^ (Supplementary Table 2).

Our findings highlight that CB/PLA electrodes made at extruder temperatures of 230 °C and 240 °C had improved electrochemical activity. This suggests there is an increased electroactive surface area as extruder temperature increases. This may be due to increases in the electrode surface area and/or due to the presence of a greater number of conductive pathways due to a reduction in the size/number of voids between print layers in electrodes printed at higher temperatures.

### Understanding the changes in structure of the electrode when printed at varying extruder temperatures

Since there was a significant difference in the electrochemical activity of electrodes made at varying extruder temperatures, we investigated if these changes were due to variations in the electrode surface by conducting capacitance and electrochemical impedance spectroscopy measurements.

**Figure 4:**
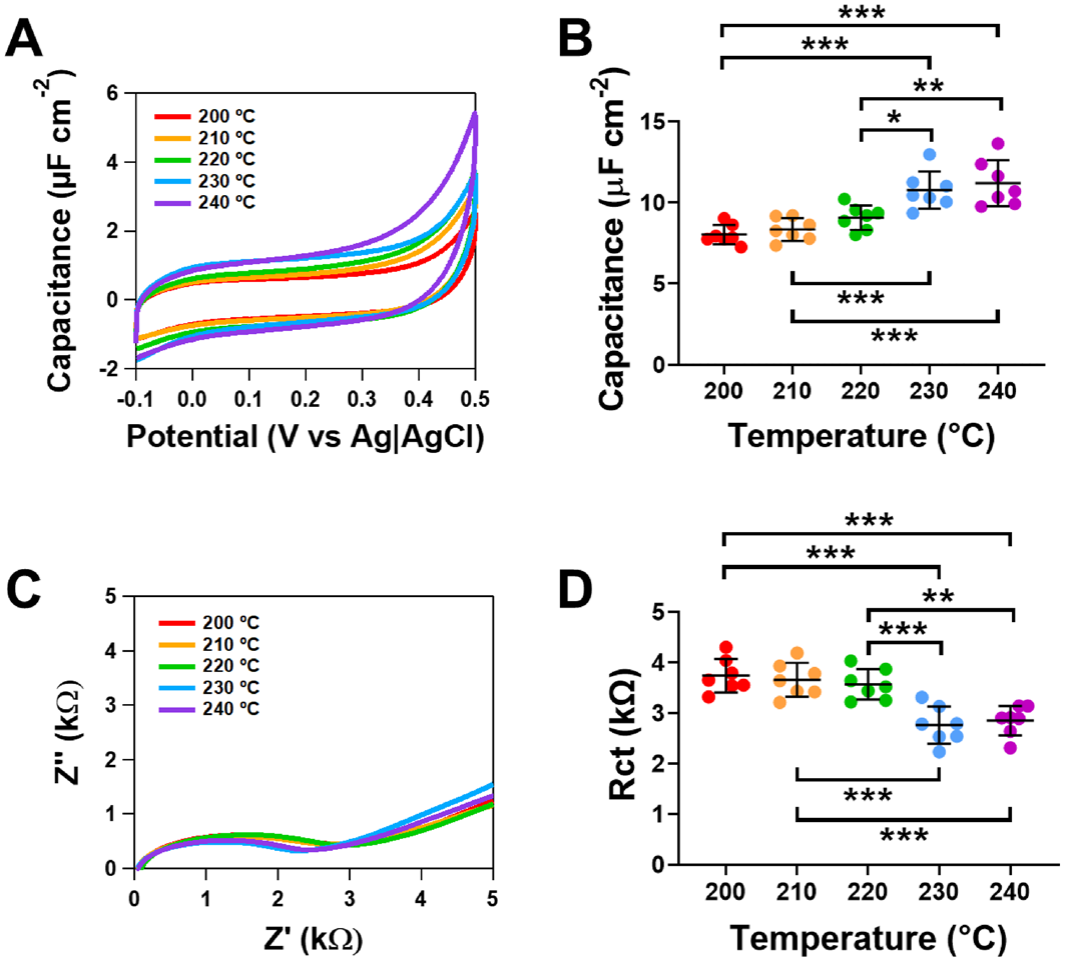
Comparing the capacitance and resistivity of 3D printed electrodes made using varying extruder temperatures where the nozzle diameter was 0.6 mm and heated bed temperature was 50 °C. (**A**) Cyclic voltammograms in 1 M KCl at 0.1 V s^−1^ (**B**) overall capacitance data for electrodes are varying printing speed. (**C**) Nyquist plots for the electrodes made at different print speeds. (**D**) Overall responses for the charge-transfer resistance (*R*ct). Data shown as mean ± SD, where n = 7, *p < 0.05, **p < 0.01 and ***p < 0.001.

As capacitance is directly proportional to the electroactive area at the electrode surface, we measured capacitance by cyclic voltammetry. Voltammograms in 1 M KCl are shown in Fig. 4A for electrodes made at different extruder temperatures. There was a significant increase in the capacitance for electrodes printed at 230 °C when compared to 200 °C (*p* < 0.001), 210 °C (*p* < 0.001) and 220 °C (*p* < 0.05, n = 7, Fig. 4B). A significant increase in the capacitance was also observed for electrodes printed at 240 °C when compared to 200 °C (*p* < 0.001), 210 °C (*p* < 0.001) and 220 °C (*p* < 0.01, n = 7). No difference in the capacitance was observed between electrodes printed at extruder temperatures of 230 °C and 240 °C (n = 7, Fig. 4B).

The Nyquist plots for the electrodes made at varying extruder temperatures are shown in Fig. 4C, where the interfacial charge-transfer resistance (R_ct_) was obtained. There was a significant decrease in the R_ct_ on electrodes printed at 230 °C when compared to 200 °C, 210 °C and 220 °C (*p* < 0.001, n = 7, Fig. 2D). A significant decrease in the R_ct_ was also observed for electrodes printed at 240 °C when compared to 200 °C (*p* < 0.001), 210 °C (*p* < 0.001) and 220 °C (*p* < 0.01, n = 7, Fig. 4D). No difference in the R_ct_ was observed between electrodes printed at extruder temperatures of 230 °C and 240 °C (n = 7, Fig. 4D). Our findings highlight that R_ct_ decreases, and capacitance increases on CB/PLA electrodes when printed at higher extruder temperatures. These findings support the voltammetric studies conducted using redox probes when exploring the impact of extruder temperature. Overall, these results suggest that when electrodes are printed at lower extruder temperatures there is a reduced electroactive surface due to differences in either the electrode surface area and/or a reduced number of conductive pathways present due to an increased number and/or size of voids between the print layers, which would make electrodes printed at lower extruder temperatures more resistive.

**Figure 5:**
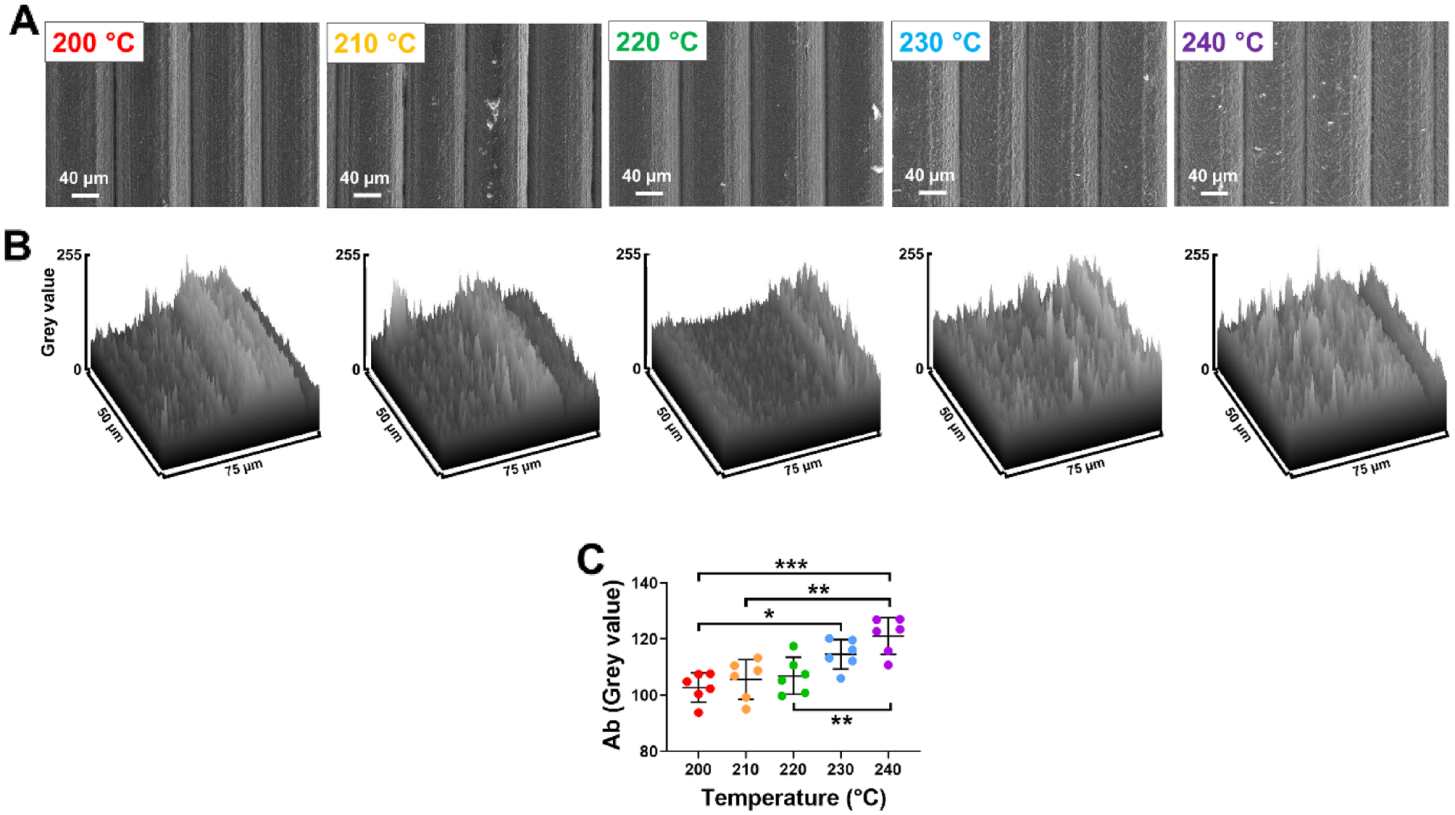
Analysis of the CB/PLA electrode surface. (**A**) Scanning electron microscopy imaging of four print layers of the CB/PLA electrode surface area made at varying extruder temperatures. (**B**) The surface profile analysis of a single print layer and (**C**) analysis of the average surface roughness (Ab) measured in grey values obtained from three print layers of the electrode. Data shown as mean ± SD, where n = 6, *p < 0.05, **p < 0.01 and ***p < 0.001.

SEM images were obtained to understand if there was a difference observed in the electrochemical activity due to variations in the electrode surface. Figure 5A shows the response of four individual print layers on the surface of the CB/PLA electrode following electrochemical pre-treatment in NaOH. No obvious visible differences were observed in the width of each print layer and depth of the convex semi-circle formed by print layers when comparing the different electrode made by different extruder temperatures. To gain further insight into the roughness of the electrode surface, an image profile analysis was conducted of the SEM image, where the responses are shown in Fig. 5B. The grey values are a measure of greyscale, in with smaller values are closer to white and larger values are closer to black. The average surface roughness (Ra) over 3 print layers of the electrode surface was obtained. There was a gradual increase in the roughness of the electrode surface with increasing extruder temperature (Fig. 5C). The was a signficant increase in the roughness of the electrode printed at extruder temperatures of 240 °C when compared to 220 °C, 210 °C (both *p* < 0.01) and 200 °C (*p* < 0.001, n = 6). There was also a significant increase in the roughness of the electrode printed at 230 °C when compared to 200 °C (*p* < 0.05, n = 6). These findings indicate that there are increases in the electrode roughness, which potentially may increase the electroactive surface area resulting in the enhanced electrochemical activity observed at higher extruder temperatures.

**Figure 6:**
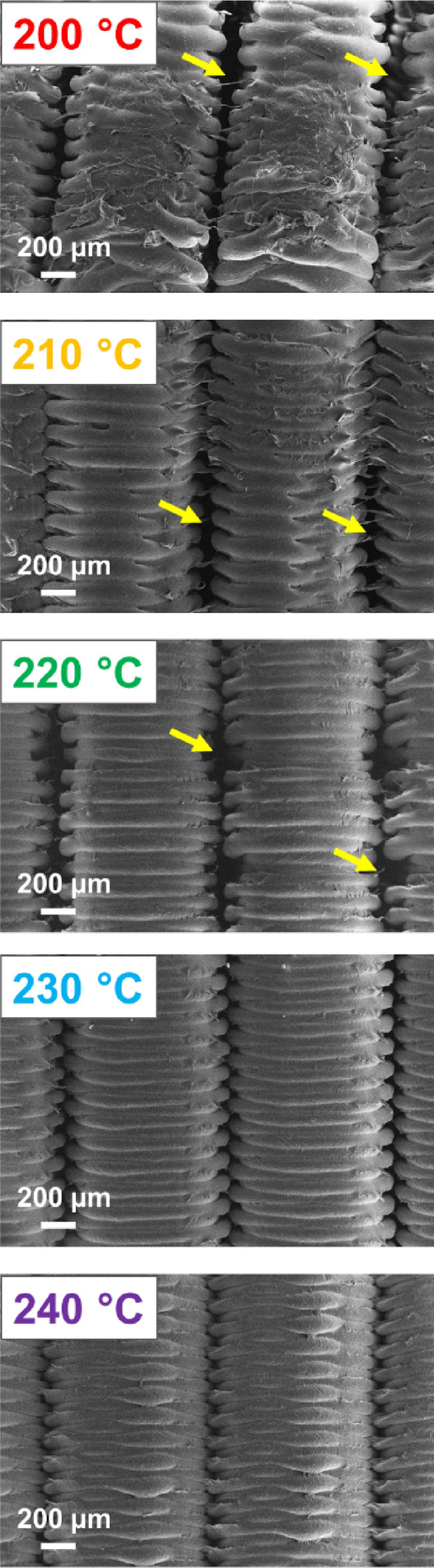
Scanning electron microscopy imaging of the internal cross-section of CB/PLA electrodes made at varying extruder temperatures. Arrows highlight the presence of voids between the printing layers within the electrode structure.

SEM images of the cross-section of the electrode were conducted to understand how the print layers that formed the electrode have adhered together (Fig. 6). When comparing the adherence between print layers there was a clear presence of voids between the print layers in electrodes printed at 220 °C and lower, with the size of these voids increasing and the structural order of the print layers reducing at lower extruder temperatures. This suggest that adhesion between print layers is reduced with lower extruder temperatures. These findings strongly indicate that the reduced electrochemical activity at lower extruder temperatures is also due to the presence of voids and poor adhesion between print layers, which in turn reduces the probability to form conductive pathways from ohmic connection to the electrode surface. These findings support those observed in other studies conducted using PLA parts, where the presence of voids between print layers was observed with lower extruder temperatures^20,31,32^.

### Exploring the impact of different 3D printers on the electrochemical activity of CB/PLA electrodes

**Figure 7:**
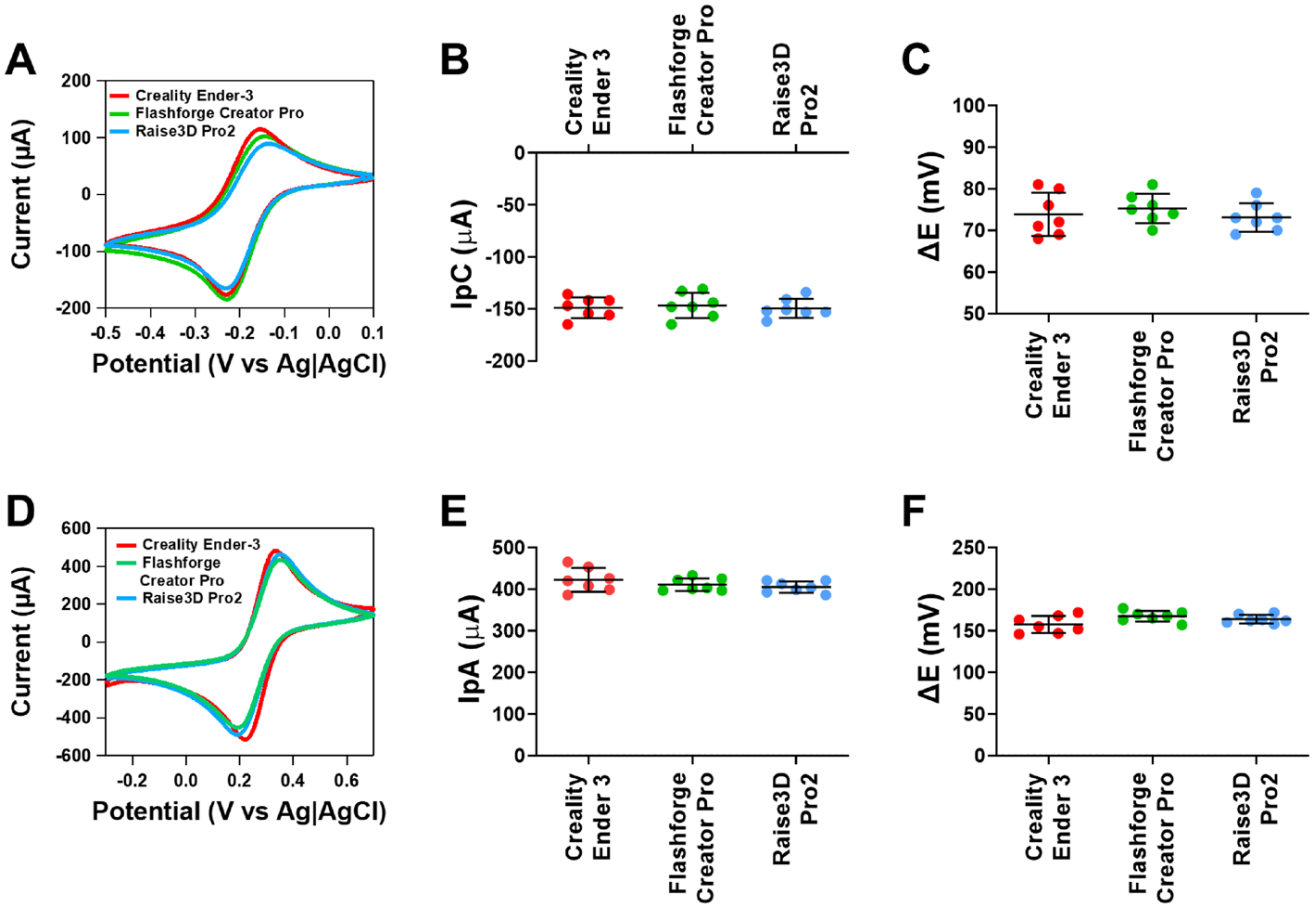
Responses of CB/PLA electrodes made using different 3D printers. (**A**) Representative cyclic voltammograms of 1 mM ruthenium hexaamine in 1 M KCl at 0.05 V s^−1^, (**B**) Cathodic peak current, (**C**) ΔE of ruthenium hexaamine (**D**) representative cyclic voltammograms of 5 mM ferricyanide in 1 M KCl at 0.05 V s^−1^, (**E**) anodic peak current and (**F**) ΔE of ferricyanide. The extruder temperature was 230 °C, heated bed temperature was 50 °C and the nozzle diameter was 0.6 mm. Data shown as mean ± SD, where n = 7.

A wide variety of 3D printers have been utilised to make printed electrodes, of which the instrument tolerances are varied^41^. Using the optimised instrumental parameters within this study, we compared three different 3D printers. The Creality Ender 3 is the cheapest and widely used by hobbyists, the Flashforge Creator Pro is a medium-end use printer and lastly the Raise3D Pro2 is a high-end use printer. Supplementary Fig. 2 showed that there were no structural differences in the print layers on the surface of the CB/PLA electrodes made using different printers. Figure 7A shows cyclic voltammograms for ruthenium hexaamine, where no differences in the responses were observed. When comparing the different 3D printers, there was no significant difference in the cathodic peak current (n = 7, Fig. 7B) and ΔE (n = 7, Fig. 7C). Figure 7D shows cyclic voltammograms for ferricyanide, where no differences in the responses were also observed. When comparing between the 3D printers, there was no significant difference in the anodic peak current (n = 7, Fig. 7E) and ΔE (n = 7, Fig. 7F). However, from the data, there was a clear difference in the precision, where the relative standard deviation was 6.7% on the Creality Ender 3 printer but reduced to 3.7% on the Flashforge Creator Pro and 3.3% on the Raise 3D Pro2. Therefore, these findings highlight that different 3D printers have no overall effect on the current or electron transfer kinetics, but higher-end printers are more likely to provide greater precision in the printing which in turn will enhance batch reproducibility.

## Conclusion

3D printing has emerged as a simple and effective approach towards the manufacture of conductive carbon electrodes for sensing applications. Studies conducted on 3D printable thermoplastics have shown that 3D printer instrument parameters can have a significant influence on the mechanical strength of the electrode. However, no studies to-date have investigated the influence of 3D printer instrument settings on the electrochemical activity of CB/PLA electrodes. Extruder temperatures of 230 °C and 240 °C enhanced the electrochemical activity of CB/PLA electrodes due to an increase in the roughness of the electrode surface and a reduction in the number of voids between print layers. Different nozzle diameters or variations in the heated bed temperature did not alter the electrochemical activity of CB/PLA electrodes. Different 3D printers did not alter the electrochemical activity of the CB/PLA electrodes, but high-end 3D printers reduced variability within a batch of electrodes. Our findings highlight that when manufacturing conductive thermoplastics using 3D printing, instrument settings should be considered to optimise the performance of the printed electrochemical sensor for analytical studies.

## Method

### Manufacture of 3D printed CB/PLA electrodes

CB/PLA filament (marketed as Proto pasta, was purchased from filaprint, UK) was used to make 3 mm height and 10 mm diameter cylinders using a Creality Ender 3 printer. For printing parameters, we used two outer perimeter shells, 100% infill, 0.1 mm print layer thickness, 60 mm/s print speed and vertical orientation. Previous studies have highlighted these parameters enhance the conductivity of CB/PLA electrodes^11,15,34^. To investigate the impact of nozzle diameter, all electrodes were printed at 230 °C extruder temperature and heated bed temperature of 50 °C, using nozzle diameters from 0.3 to 0.6 mm. To understand the impact of the heated bed temperature, electrodes were printed at 50 °C and 70 °C, where the extruder temperature was 230 °C and the nozzle diameter was 0.6 mm. To explore the influence of extruder temperature, cylinders were made at temperatures of 200–240 °C. This range was chosen as this was indicated as the working range of CB/PLA filament by the manufacturer. The nozzle diameter was 0.6 mm and the heated bed temperature was 50 °C. To compare between 3D printers, electrodes were made using 0.6 mm nozzle diameter, 50 °C heated bed temperature and 230 °C extruder temperature on each machine. Alongside the Creality Ender 3, we utilised Flashforge Creator Pro and Raise3D Pro2 printers.

As previously shown^18^ electrical connection was made by attaching a copper wire using conductive silver epoxy (CircuitWorks) to the CB/PLA cylinders. This was then sealed using a glue gun to form an insulation around the electrode to expose only the disc of the cylinder. Supplementary Fig. 1 provides a schematic highlighting out approach to making CB/PLA electrodes and photographs of the final electrode utilised for electrochemical investigations.

### Scanning electron microscopy (SEM)

SEM measurements were carried out based on a previously published approach^18^. Briefly, the CB/PLA electrodes were imaged using a Zeiss SIGMA field emission gun SEM equipped with an Everhart–Thornley detector operating in secondary electron detection mode, using 5 kV accelerating voltage, a 20 µm aperture, and 8.1 mm working distance. The surface of the electrodes was imaged following electrochemical pre-treatment in NaOH. To investigate the cross-section of the electrode structure of the electrode a 1 cm CB/PLA cube was printed without any outer perimeter shells.

### Electrochemical characterisation of the 3D printed electrodes

A three-electrode system was used to conduct electrochemical measurements, where the counter electrode was a platinum wire, the reference electrode was Ag|AgCl (3 M KCl) and the working electrode was our various 3D-printed CB/PLA electrodes. To conduct electrochemical experiments, a CH 760E potentiostat (CH instruments, Texas) was used.

Prior to conducting experimental studies, electrochemical pre-treatment was performed on electrode surfaces in 0.5 M NaOH by holding the potential at + 1.4 V vs Ag|AgCl for 200 s and then at − 1.0 V vs Ag|AgCl for 200 s^6,7^.

Measurements were conducted in 1 mM ruthenium hexaamine in 1 M KCl where the potential window used was 0.1 V to − 0.5 V vs Ag|AgCl. For studies in 5 mM ferricyanide in 1 M KCl, the potential window was + 0.7 to − 0.3 V vs Ag|AgCl for ferricyanide. All experiments were performed using a scan rate of 50 mV/s.

### Determination of electrode capacitance and resistance

Electrochemical Impedance Spectroscopy (EIS) measurements were performed to obtain the charge transfer resistance. Measurements were carried out in 0.5 mM potassium ferricyanide and 0.5 mM potassium ferrocyanide in 1 M KCl at a potential equal to the anodic peak potential. A frequency range of 100 kHz to 0.01 Hz and an amplitude of 5 mV were used. Capacitance was measured in 1 M KCl at 100 mV/s in the potential window of − 0.1 to + 0.5 V vs Ag|AgCl and calculations were conducted at 0.3 V.

### Data analysis

The cyclic voltammetry measurements were analysed for anodic/cathodic peak potential, the difference between the anodic and cathodic peak potential (ΔE) and anodic/cathodic peak current using CHI 760E software (CH instruments, Texas). To measure the capacitance, the mean difference in anodic and cathodic current (Δi) at 0.3 V was divided by two times the scan rate (2*v*). This was then normalised by the geometric surface area of the electrode which was 0.785 cm^2^^42^.

To understanding differences in the roughness of the CB/PLA electrode, an image profile analysis was conducted using Image J 1.53e software (NIH, USA), in which the surface profile of the electrode surface was obtained as grey values. The average surface roughness (Ra) over 3 print layers of the electrode surface was obtained and compared between electrodes made by different instrument settings. Data was shown as mean ± the standard deviation (SD). Statistical analysis (GraphPad Prism 9.0) was conducted using student t-tests and two-way ANOVA with Sidak post hoc tests.

## Supplementary Information

Supplementary Information.

## Data Availability

The datasets used and analysed during the current study available from the corresponding author on reasonable request.
