# Supplementary material for: Influence of instrument parameters on the electrochemical activity of 3D printed carbon thermoplastic electrodes
Source: Sci Rep. 2023 Jan 7;13:339. doi: 10.1038/s41598-023-27656-7 (PMC9825385; doi:10.1038/s41598-023-27656-7)

**Supporting Information**

**Influence of instrument parameters on the electrochemical activity of 3D printed carbon thermoplastic electrodes**

Ricoveer Singh Shergill^1, 2^, Chloe L. Miller^1, 2^ and Bhavik Anil Patel ^1, 2*^

^1^School of Applied Sciences, ^2^Centre for Stress and Age-Related Disease, Brighton, UK, BN2 4GJ.


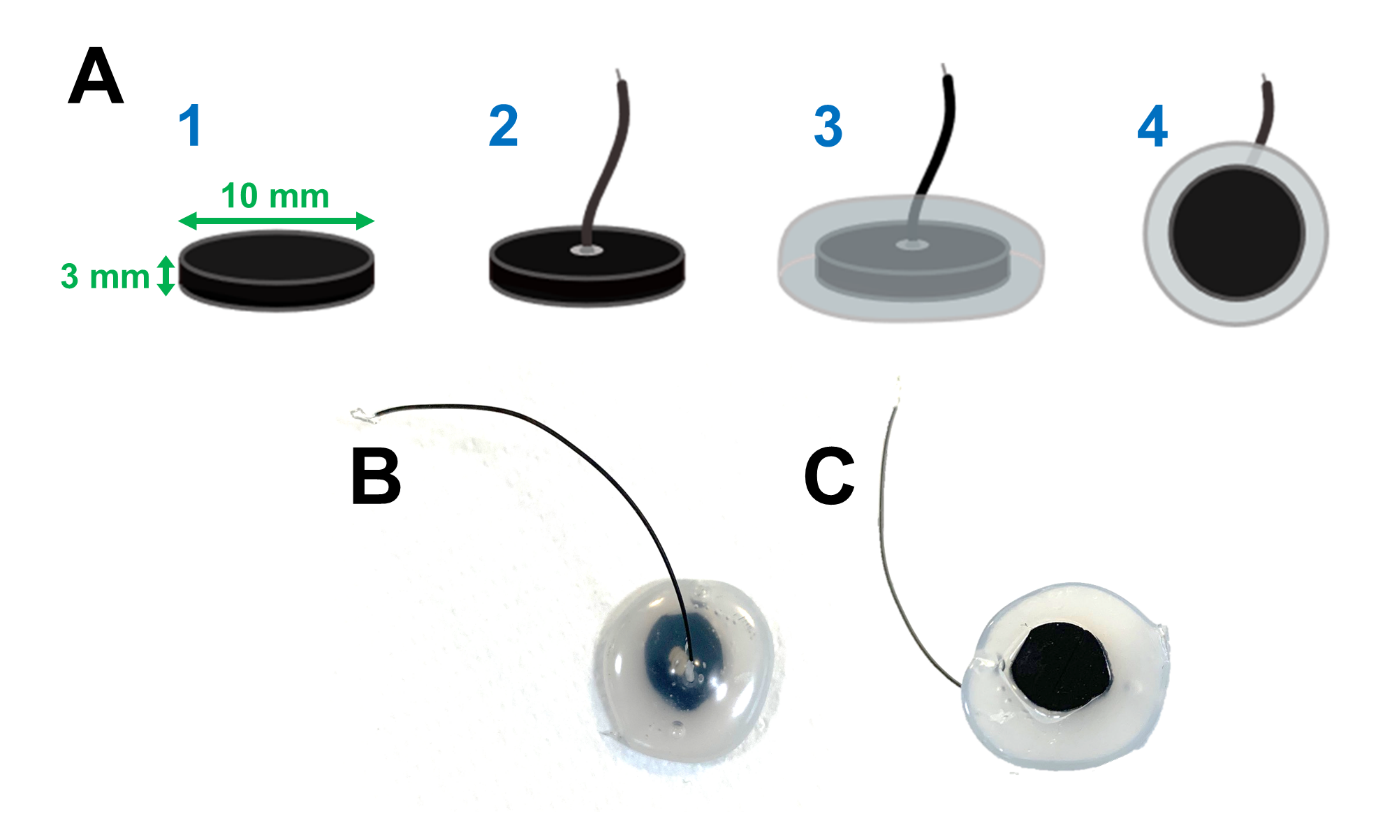


**Supplementary Figure 1.** Manufacturing of 3D printed CB/PLA electrodes. (A) Schematic showing the process used to manufacture the CB/PLA electrode. Where (1) shows the printed 10 x 3 mm 3D printed CB/PLA cylinder, (2) ohmic connection made by attaching a silver wire using silver epoxy, (3) the electric conduction is sealed using a glue gun to insulate the electrode in which (4) only the disc of the electrode is exposed. (B) Photograph of the final electrode showing a view from the top and (C) a view showing the active surface area of the electrode.

**Supplementary Table 1.** Active surface area of the CB/PLA electrodes made at varying extruder temperatures using the Randles–Ševčík equation. Measurements calculated from experiments carried out using 1mM ferricyanide in 1 M KCl. % Relative standard deviation (RSD) is shown in the brackets. The value in green provides the percentage of the geometric surface area.

| Extruder temperature (°C) | Electroactive surface area |
| --- | --- |
| 200 | 0.106 ± 0.01 cm^2^  **13.6 ± 1.9 %** |
| 210 | 0.107 ± 0.01 cm^2^  **13.7 ± 1.7 %** |
| 220 | 0.111 ± 0.02 cm^2^  **14.2 ± 1.9 %** |
| 230 | 0.157 ± 0.01 cm^2^  **20.0 ± 1.6 %** |
| 240 | 0.149 ± 0.02 cm^2^  **18.9 ± 1.9 %** |

**Supplementary Table 2.** Heterogenous electron transfer kinetics (HET, kº) based on the method by Nicholson. Data shown for electrodes made at varying extruder temperatures using 1 mM potassium ferrocyanide in 1 M KCl. % Data shown as mean ± SD., n=7.

| Extruder temperature (°C) | HET, kº |
| --- | --- |
| 200 | 3.2 × 10^-5^ ± 1.1 × 10^-5^ cm s^-1^ |
| 210 | 2.2 × 10^-5^ ± 1.3 × 10^-5^ cm s^-1^ |
| 220 | 3.5 × 10^-5^ ± 1.3 × 10^-5^ cm s^-1^ |
| 230 | 5.9 × 10^-5^ ± 9.3 × 10^-6^ cm s^-1^ |
| 240 | 5.1 × 10^-5^ ± 6.8 × 10^-6^ cm s^-1^ |

**Supplementary Figure 2.** Scanning electron microscopy imaging of four print layers of the CB/PLA electrode surface area made using different 3D printers.


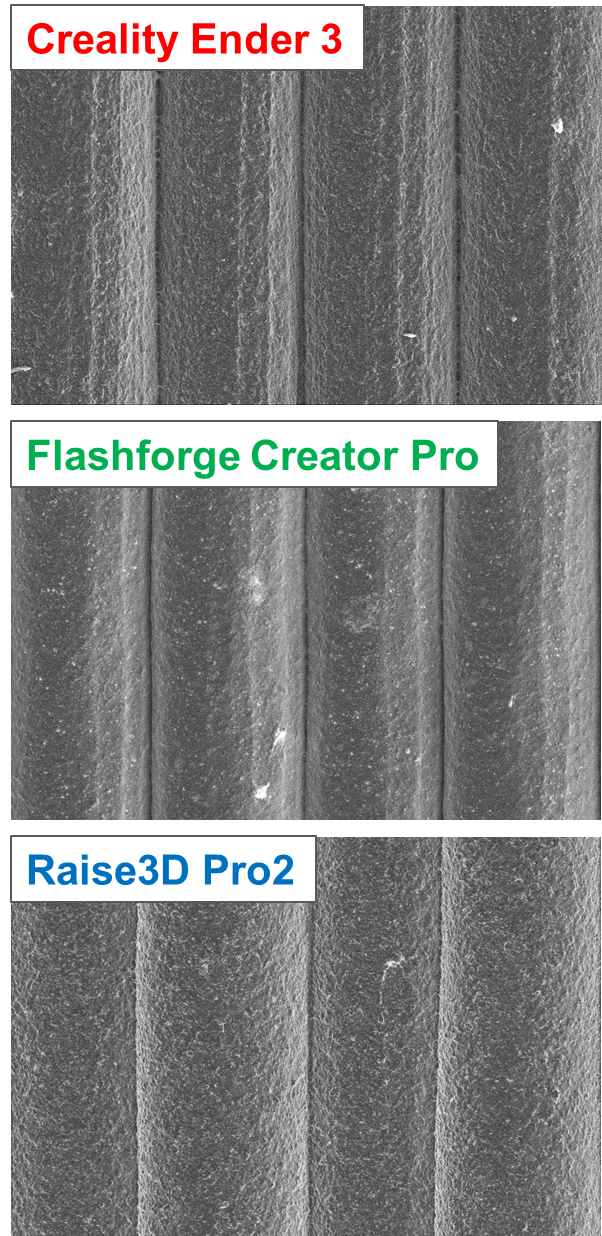

Supplement: Supplementary file 1 — Supplementary Information. [file 41598_2023_27656_MOESM1_ESM.docx]
